# Isolation of multiple plant growth-promoting fungi and their effect on rice growth improvement on non-grain converted land

**DOI:** 10.3389/fpls.2025.1618073

**Published:** 2025-08-13

**Authors:** Xuqing Li, Xiaoxu Ren, Han Chen, Yukang Xin, Tiefeng Zhou, Jianli Yan, Jun Xu, Munazza Ijaz, Temoor Ahmed, Bin Li, Qurban Ali

**Affiliations:** ^1^ Institute of Vegetable, Hangzhou Academy of Agricultural Sciences, Hangzhou, China; ^2^ State Key Laboratory of Rice Biology and Breeding, Ministry of Agriculture and Rural Affairs Key Laboratory of Molecular Biology of Crop Pathogens and Insect Pests, Zhejiang Key Laboratory of Biology and Ecological Regulation of Crop Pathogens and Insects, Zhejiang Engineering Research Center for Biological Control of Crop Pathogens and Insect Pests, Institute of Biotechnology, Zhejiang University, Hangzhou, China; ^3^ Institute for Plant Protection and Fertilizer, Agricultural Technology Extension Center of Fuyang District, Hangzhou, China; ^4^ Department of Life Sciences, Western Caspian University, Baku, Azerbaijan; ^5^ Xianghu Laboratory, Hangzhou, China; ^6^ Department of Biology, College of Science, United Arab Emirates University, Al-Ain, United Arab Emirates

**Keywords:** non-grain converted lands, plant growth promoting fungi, identification, soil properties, microbial community

## Abstract

**Introduction:**

Land cultivation is the cornerstone of national food security. However, with the development of non-grain production on cultivated land, China has to use less cultivated land to feed a larger population of the world. To effectively resolve issues caused by non-grain production on cultivated land, Zhejiang Province has initiated efforts to restore non-grain-converted land back to grain cultivation. Whereas, the discovery and application of plant growth-promoting fungi (PGPF) can offer promising solutions to these challenges.

**Methods:**

PGPF was isolated and identified from soil converted from non-grain lands based on bioassays for plant growth promoting traits, and then their impacts on soil properties and microbial community structure were also investigated.

**Results:**

In this study, 15 fungal isolates from 108 soil samples were considered as potential PGPF due to their ability to solubilize phosphate (11.91 to 31.65 mm), produce both siderophores (17.09 to 24.66 mm) and indole-3-acetic acid (8.79 to 50.23 μg/mL or 36.72 to 96.50 μg/mL). Results of in vivo assays showed that isolates TL-B31f and FY-R41f could cause a great increase in plant height (15.30% and 13.84%), root length (33.62% and 43.31%), seedling fresh weight (78.58% and 89.77%) and dry weight (9.31% and 28.12%) of rice compared to the control. Based on morphological and molecular analyses, isolates TL-B31f and FY-R41f were identified as Aspergillus tubingensis and Talaromyces veerkampii, respectively. Furthermore, after 55 days of inoculation with the two isolates, the soil content of available phosphate was significantly increased by 42.52% and 48.51%, respectively, compared to the control. In addition, high-throughput sequencing analysis showed that compared with the control, the microbial community composition of the two isolates treatments was reconstructed by increasing or decreasing some specific microbes, while soil properties, such as pH, soil organic matter (SOM), total phosphorus (TP), and available phosphate (AP) might play important roles in modulating rice growth by influencing the composition of microbial communities.

**Conclusions:**

Overall, our findings highlight the potential of these isolates to be developed into novel biofertilizers for crop growth in non-grain lands.

## 1 Introduction

Rice (*Oryza sativa* L.) is the critical staple food for nearly half the world’s population, and global rice consumption is projected to reach about 550 million tons by 2030 (Yuan et al., 2021). Maintaining a steady increase in rice production is vital to ensure social stability and sustainability (Hashim et al., 2024). However, with rapid socio-economic development, dietary patterns in China have partially transformed from rice toward diversified diets with more energy and macronutrients (Yuan et al., 2019). To accommodate this change and obtain more financial benefits, lots of cultivated lands have been used for non-grain production, such as planting fruits, vegetables, bamboo, etc., in recent years (Zhang et al., 2024). Nevertheless, excessive non-grain production on cultivated land not only seriously threatens national grain security, but also triggers a series of problems, such as soil quality and ecosystem degradation due to heavy chemical inputs (Yang and Zhang, 2021; Zhu et al., 2022b; He et al., 2024). To solve these problems, Zhejiang Province has initiated efforts to restore non-grain converted land back to grain cultivation. During this conversion process, soil health is critical in determining agricultural productivity, ecosystem health, and sustainable development.

Among many factors contributing to healthy soil, microbes stand out as an important part of the soil ecosystem by improving soil structure and fertility, driving nutrient cycling, suppressing soil-borne disease, and supporting plant growth (Wang et al., 2023; Chen et al., 2024; Romão et al., 2025). Among the diverse microbes, PGPF have attracted more attention in recent years. Varieties of PGPF belonging to genera *Aspergillus*, *Arbuscular*, *Fusarium*, *Penicillium*, *Phoma*, *Podospora*, and *Trichoderma* have been widely studied (Hossain and Sultana, 2020; Adedayo and Babalola, 2023). PGPF have been found to be able to promote crop growth and improve soil conditions through mechanisms such as phosphate solubilization (Chuang et al., 2007; Radhakrishnan et al., 2015; Li et al., 2016), organic matter mineralization (Yadav et al., 2009), production of plant growth-promoting hormones and enzymes (Abou Alhamed and Shebany, 2012; Sofo et al., 2012), generation of volatile organic compounds (Naznin et al., 2013; Bitas et al., 2015), and induction of disease resistance (Murali et al., 2013; Murali and Amruthesh, 2015; Naziya et al., 2019). Plant growth promotion processes by PGPF are often complex, and arrays of interconnected mechanisms are involved in these processes, which help PGPF maintain rhizosphere competence and stability in performance (Hossain and Sultana, 2020). Thus, it is important to find novel PGPF that can be used as bioinoculants with multiple traits for improving plant growth and soil fertility in non-grain converted lands.

Besides the direct effects on plant growth, PGPF can also change the physiological features of soil bacteria (Akinola and Babalola, 2020). Bacteria and fungi in soil generally account for >90% of total soil microbes, and soil microbial communities play several important roles in preserving ecosystem function and soil health (Banerjee and van der Heijden, 2023). Previous research has shown that *Aspergillus brunneoviolaceus* HZ23 can significantly increase the richness of bacteria in the rhizosphere soil of pakchoi in newly reclaimed land (Li et al., 2023). Soil microbial diversity plays an important role in resisting and restoring degraded ecosystems (Pedrinho et al., 2024). Diversity and asynchrony in soil microbial communities stabilize ecosystem functioning (Wagg et al., 2021). However, the response patterns of bacterial and fungal communities to agricultural practices and environmental factors may differ, thus leading to variations in their ecological functions (Zhou et al., 2025). For example, bacteria not only exhibited greater sensitivity to fire disturbance than fungi, but also recovered faster than fungi following one month of burning (Arunrat et al., 2024). Soil bacteria are also more sensitive than fungi to the fertilization practices, while fungi are more active in response to crop conversion from wheat-maize to wheat-soybean rotation (Ai et al., 2018). Converting upland with maize to paddy with rice fields alters soil nitrogen (N) microbial functions at different depths in the black soil region (Li et al., 2024). Yet, soil ecosystem multifunctionality is strongly linked with crop yield and environmental sustainability (Deng et al., 2024). Therefore, it is essential to investigate further how different PGPF affect bacterial and fungal communities of rice in non-grain converted land after inoculation, as well as their respective ecological roles in soil health. However, at present, a limited number of researches are available on PGPF improving the soil conditions of non-grain converted lands and enhancing their transformation back to grain production.

In order to enhance soil fertility of typical non-grain converted lands, this study was carried out to isolate fungal isolates from non-grain converted lands, screen novel PGPF by evaluating their plant growth promoting traits, and identify them based on combined analysis of morphological and molecular data. Furthermore, the potential of the obtained PGPF to be developed into novel microbial control products was determined by investigating their effect on soil properties and microbial community structure of rice in non-grain converted land and clarifying their respective ecological roles in soil health.

## 2 Methods

### 2.1 Fungal isolation

One hundred and eight soil samples were collected with a hand auger from a 5–20 cm soil layer around the crown of plants in Jiande (loquat-rice), Chun’an (mulberry-rice), Tonglu (blueberry-rice), Fuyang (grape-rice), Lin’an (bamboo-rice), and Yuhang (seedling-rice), Zhejiang province, China. For each conversed mode, six samples were taken from each field (such as loquat-rice converted mode in Jiande, every six samples were collected from loquat garden, paddy field converted from loquat garden, and perennial paddy field, respectively) using the five-point sampling method (Panagos et al., 2014), and a total of eighteen samples were collected. Then, each sample was packed individually on-site into a sterilized and sealed polythene bag, and transported to the laboratory using a portable cooler for further analysis. Indeed, after dissolving 10 g fresh soil in 90 mL sterile water, mixing well by vortex, and serially diluting with 9 mL of sterile water, 100 μL of 10^–3^ dilution was inoculated on potato dextrose agar (PDA, potato extract 200 g, dextrose 20 g, agar 20 g, ddH_2_O 1000 mL) medium and then incubated at 25°C for 3–5 days. After three times of repetitive purification on PDA medium, the colonies with different morphological characteristics and higher growth rate were selected to further purify via single-spore isolation, and then stored in 20% glycerol at -70°C.

### 2.2 Identification of fungal isolates

The fungal isolates were identified as described before (Samson et al., 2014; Tian et al., 2021), which was carried out by incubating them on PDA, Czapek yeast autolysate agar (CYA, czapek concentrate 10 mL, sucrose 30 g, yeast extract 5 g, K_2_HPO_4_1 g, CuSO_4_·5H_2_O 0.005 g, ZnSO_4_·7H_2_O 0.01 g, agar 20 g, ddH_2_O 1000 mL), and male extract autolysate (MEA, malt extract 50 g, CuSO_4_·5H_2_O 0.005 g, ZnSO_4_·7H_2_O 0.01 g, agar 20 g, ddH_2_O 1000 mL) medium for 7 days at 25°C, and observing colony morphology of each isolate. Molecular identification of the fungal isolates was carried out using the ITS, TUB, CaM, and RPB2 combined dataset with the following steps: mycelia of each test isolate were harvested from the surface of PDA, frozen in liquid nitrogen, and extracted for genomic DNA using the Rapid Fungi Genomic DNA Isolation Kit (Sangon Biotech Co., Ltd., Shanghai, China).

Primer sets ITS5/ITS4 (5’-TCC TCC GCT TAT TGA TAT GC-3’ and 5’-GGA AGT AAA AGT CGT AAC AAG G-3’), Bt2a/Bt2b (5’-GGT AAC CAA ATC GGT GCT GCT TTC-3’ and 5’-ACC CTC AGT GTA GTG ACC CTT GGC-3’), CMD5/CMD6 (5’-CCG AGT ACA AGG ARG CCT TC-3’ and 5’-CCG ATR GAG GTC ATR ACG TGG-3’), and 5F/7CR (5’-GAY GAY MGW GAT CAY TTY GG-3’ and 5’-CCC ATR GCT TGY TTR CCC AT-3’) were used for amplification of rDNA ITS, TUB, CaM, and RPB2 genes, respectively (Hong et al., 2005; Nasri et al., 2015; Tomaha et al., 2020; Haituk et al., 2021). PCR amplification reaction mixture (50 μL) included ddH_2_O (18 μL), 2 × Hieff^®^ PCR Master Mix (25 μL), 10 μM each primer (2 μL), and DNA (3 μL). Then, PCR was carried out as follow: for ITS gene, 94°C 5 minutes; 94°C 45 s, 52°C 45 s, 72°C 60 s, 35 cycles; 72°C 10 minutes; for TUB and CaM genes, 94°C 5 minutes; 94°C 60 s, 55°C 60 s, 72°C 90 s, 35 cycles; 72°C 10 minutes; for RPB2 gene, 94°C 5 minutes; 94°C 30 s, 51°C 30 s, 72°C 60 s, 5 cycles; 94°C 30 s, 49°C 30 s, 72°C 60 s, 5 cycles; 94°C 30 s, 47°C 30 s, 72°C 60 s, 30 cycles; 72°C 10 minutes.

PCR products were visualized on 1.0% agarose gels, then purified and submitted to Tsingke Biotechnology Co., Ltd. (Hangzhou, China) for sequencing in both directions. Sequences for each region were assembled and edited using DNASTAR Lasergene (v7.0.1) (DNAStar Inc., Madison, USA) and BioEdit (v7.0.9) (North Carolina St. University, Raleigh, USA) (Tippmann, 2004), and then analyzed by BLAST search in the GenBank database. After the reference sequences of closely related species for test strains were downloaded from the GenBank database, maximum likelihood (ML) phylogenetic trees were constructed using Mega 7.0 (Kumar et al., 2016). Bootstrap replicates were performed 1000 times, and bootstrap values above 50% were indicated on the cladogram to indicate the significance of the separation.

### 2.3 Bioassays for plant growth promoting traits

The ability of the selected isolates to solubilize phosphate was evaluated in National Botanical Research Institute’s phosphate (NBRIP, glucose 10 g, Ca_3_(PO_4_)_2_5 g, MgCl_2_5 g, KCl 0.2 g, MgSO_4_·7H_2_O 0.25 g, (NH_4_)_2_·SO_4_ 0.1 g, agar 25 g, ddH_2_O 1000 mL, pH 7.0) medium (Abdallah et al., 2019). In brief, a 6-mm agar disc of test fungus was placed on NBRIP medium at 25°C for 3 days, the phosphate solubility was assessed by observing the formation of a clear halo zone surrounding the colony, and measuring the size of the phosphate-solubilizing zone. Each fungal isolate was performed in triplicate.

Fungal isolates with high phosphate solubility (producing a clear halo zone surrounding the colony) were further used to evaluate their siderophore-producing ability, which was carried out using the chrome azurol S (CAS) assay as previously described (Murugappan et al., 2011; Li et al., 2021). In brief, a 6-mm agar disc of the test fungus was placed on CAS-agar medium and incubated at 25°C for 3 days. Colonies of siderophore-producing fungus were surrounded by an orange halo in the CAS plate. Each fungal isolate was performed in triplicate.

Fungal isolates with high phosphate solubility (producing a clear halo zone surrounding the colony) and siderophore-producing (producing an orange halo surrounding the colony) ability were further analyzed for indole acetic acid (IAA) production, which was performed as described before (Li et al., 2021). In brief, a fungal piece (6 mm in diameter) was separately added to a test tube containing 10 mL PDB (PDA without agar) containing 0.1% or 1.0% tryptophan, respectively, while PDB without tryptophan was used as a control. After shaking at 25°C, 150 rpm for 7 days, approximately 2 mL of culture solution was collected and centrifuged at 4°C, 8000 rpm for 5 minutes, respectively. Finally, 1 mL of supernatant for each isolate was taken and separately mixed with 4 mL Salkowski’s reagent (0.5 M FeCl_3_, 15 mL, H_2_SO_4_, 300 mL, ddH_2_O, 500 mL). After the mixtures were incubated at room temperature for 20 minutes in the dark, the observation of pink–red color changes indicated IAA production. Absorbance was read at 530 nm using a spectrophotometer (Perkin Elmer Lambda35, Waltham, MA, USA), and IAA concentrations were calculated on the basis of the standard curve prepared by standard IAA solutions (0, 10, 20, 30, 40, 50, 100 μg/mL). Each experiment was performed in triplicate.

Plant growth promotion (PGP) activity of the selected fungal isolates was determined by evaluating their effect on growth of rice cv. Xiushui131 (purchased from Jiaxing Academy of Agricultural Sciences, China). In details, after sterilization with 75% alcohol for 1 minutes, rinsing three times with sterile water, and pre-germination on wet sterile filter papers at 25°C for two days, rice seeds were sowed into planting pots (8 cm × 8 cm × 12.5 cm) containing soil previously planted with vegetables, and then put in a greenhouse with a relative humidity of 70% and temperature of 25°C. Two days after sowing, 10 mL conidial suspension of each tested isolate (prepared by scraping the conidial masses on a 7-day-old culture grown on PDA at 25°C to sterile distilled water, and diluting to 10^6^ spores/mL) was poured into the soil surrounding the plants. Plants irrigated with 10 mL sterile water were used as controls. After 55 days of inoculation, the PGP ability was determined by measuring the seedlings height, root length, fresh and dry weight (dried in an oven at 65°C for two days), and the growth promotion efficacy (GPE) was calculated using the formula: GPE% = (treatment – control)/control × 100%. Each treatment consisted of three replicates with four pots (six plants per pot) per replicate (total 72 plants per treatment).

### 2.4 Impacts of PGPF on soil properties and microbial community structure

At harvest of rice, about 1.0 kg of soil from each treatment was used to detect soil pH, SOM, TP, and AP as previously described. Indeed, after drying at room temperature and passing through a 0.45-mm sieve, soil pH was measured using a pH meter (FE28, Mettle-Toledo, Zurich, Switzerland); SOM content was measured by the potassium dichromate volumetric method; TP was determined by automated colorimetric analysis after persulfate digestion; AP was measured by molybdenum-antimony anti-spectrophotometry following extraction with hydrochloric acid ammonium fluoride (Jackson, 1973; Brookes et al., 1985; Vance et al., 1987; Baran et al., 2019).

Simultaneously, 10 g of root-zone soil around rice plants was sampled from each treatment and stored it at -80°C for further genome sequencing. In detail, genomic DNA was extracted from 1 g of soil samples using the E.Z.N.ATM Mag–Bind Soil DNA Kit (Omega, USA) following the manufacturer’s instructions, and then the DNA quality was assessed using a Qubit 4.0 (Thermo, USA). Here, the V3-V4 region of bacterial 16S rRNA genes and the ITS1 region of fungal ITS genes were amplified using the universal primers 341F (5′−CCT ACG GGN GGC WGC AG−3′) and 806R (5′−GGA CTA CHV GGG TWT CTA AT−3′) (Wu et al., 2015), and ITS1F (5′−CTT GGT CAT TTA GGA AGT AA−3′) and ITS2 (5′−GCT GCG TTC TTC ATC GAT GC−3′) (Adams et al., 2013), respectively. PCR mixture consisted of 2×Hieff^®^ Robust PCR Master Mix (15 μL), 10 μM universal primer (1 μL of each primer), DNA template (1 μL), and ddH_2_O (12 μL). PCR thermal protocol consisted of an initial 3 minutes denaturation step at 94°C, 25 amplification cycles of 94°C for 30 s, 55°C for 30 s, 72°C for 30 s, and a final extension step of 5 minutes at 72°C. PCR products were purified using Hieff NGS™ DNA selection beads (Yeasen, China), and then pooled in equimolar concentrations and sequenced using the 2 × 250 bp pair-end sequencing protocol on an Illumina MiSeq system (Sangon Biotechnology Co., Ltd., Shanghai, China).

After sequencing, raw data were assembled using PEAR (v0.9.8) and preprocessed using PRINSEQ to remove low-quality reads (average quality score < 20) to ensure high data quality (Schmieder and Edwards, 2011; Zhang et al., 2014). After primers were trimmed with Cutadapt (v1.18), clean reads were clustered into operational taxonomic units (OTUs) of ≥97% similarity using Usearch (v11.0.667) (Martin, 2011; Edgar, 2013, 2016). After selection of the representative read of each OTU using the QIIME package (v2020.06), all bacterial and fungal OTU representative sequences were classified taxonomically by blasting against the RDP database and UNITE database, respectively (Altschul et al., 1997; Wang et al., 2007; Caporaso et al., 2010; Quast et al., 2013; Urmas et al., 2013). Among them, relative abundances (RAs) of *Aspergillus* and *Talaromyces* species, and their relationships with environmental factors were especially focused using Origin software (v2023, Hampton, USA) and redundancy discriminant analysis (RDA), as well as correlation network.

### 2.5 Statistical analysis

One-way variance analysis (ANOVA) was performed using SPSS (v16, SPSS, USA). OTUs and alpha diversity indices (Chao1 and Shannon index) were analyzed by Origin software. The difference of microbial communities among different samples was measured by principal component analysis (PCA) based on beta diversity metrics from the Bray-Curtis metrics (Ramette, 2007), and the significant difference was tested using permutational multivariate ANOVA (PERMANOVA) with 999 permutations used to calculate *p*-values (Dixon, 2003). RAs (at the phylum and genus level, respectively) and heat map (at the family level) of the dominant microbes were calculated using Origin software. Differential biomarkers between groups were discovered by linear discriminant analysis effect size (LEfSe) (Segata et al., 2011). Different environmental factors on microbial community structure were calculated using RDA. Diagrams of the correlation network between soil properties and microbial taxa were performed using R software (v4.1.3).

## 3 Results

### 3.1 Assessment of plant growth promoting traits

**Figure 1:**
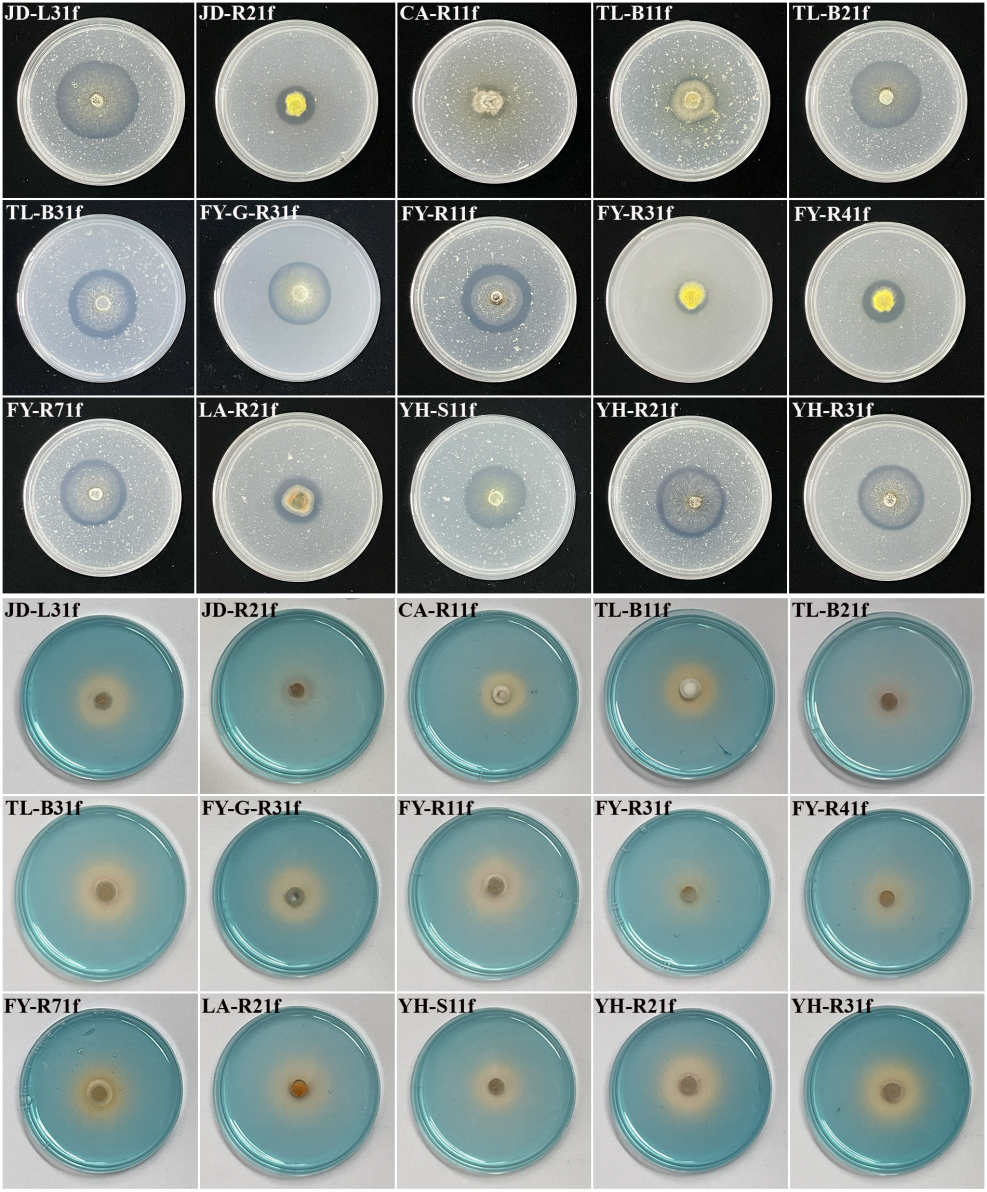
Assessment of phosphate solubilization and siderophore production ability of 15 fungal isolates on NBRIP medium (upper), and on CAS medium (lower), respectively.

**Figure 2:**
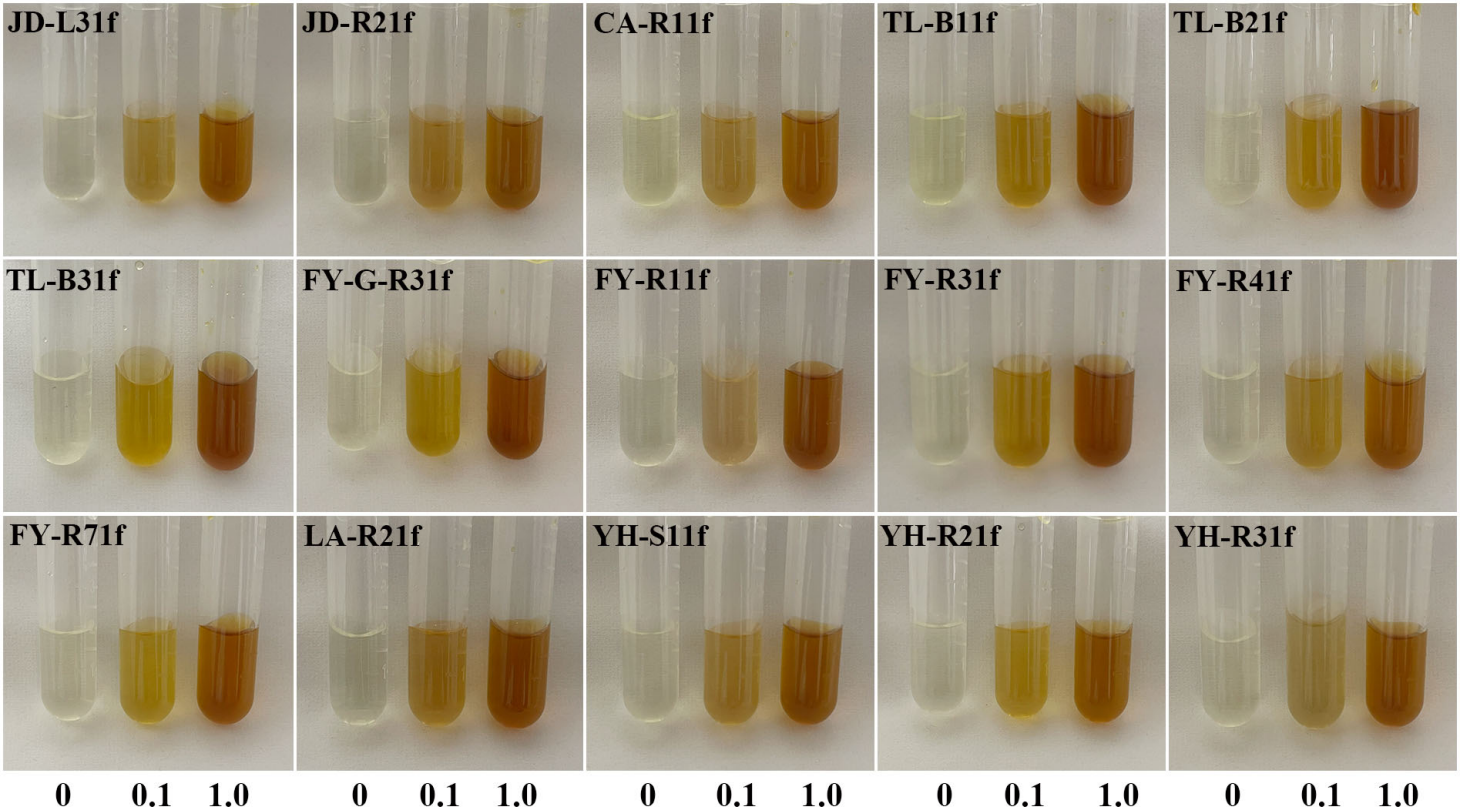
Ability of 15 fungal isolates to produce IAA in PDB medium amended with 0.1% or 1.0% tryptophan.

The isolated fungi were screened to determine their plant growth-promoting traits. Results showed that 15 isolates displayed the ability of phosphate solubilization, siderophore production, and IAA production (**Figures 1**, **2**). Indeed, as shown in **Table 1**, the diameter of the phosphate-solubilizing halo ranged from 11.91 to 31.65 mm, among which, isolates JD-L31f (31.65 mm), TL-B21f (28.96 mm), and FY-R71f (28.43 mm) exhibited the highest phosphate solubilization ability (**Figure 1** upper). Moreover, the siderophore production ability was tested by growth of fungal isolates on CAS media. Results showed that all 15 isolates could secrete iron carriers. Among them, isolates YH-R21f, TL-B31f, LA-R21f, and YH-R31f displayed the siderophore-containing area of 24.66, 24.41, 24.16, and 23.66 mm, respectively, which were significantly (*p* < 0.05) higher than those of the other isolates (**Figure 1** lower). Finally, all isolates had the ability to produce IAA in PDB medium amended with 0.1% or 1.0% tryptophan. At 0.1% tryptophan, the highest IAA production was obtained in isolates TL-B21f (50.23 μg/mL) and YH-R21f (31.18 μg/mL), while at 1.0% tryptophan, the highest IAA production was achieved in isolates FY-G-R31f (96.50 μg/mL) and TL-B31f (95.29 μg/mL) (**Figure 2**).

**Table 1:** Assessment of the growth promotion ability of 15 selected fungal isolates.

Strains	Phosphate solubilization (mm)	Siderophore production (mm)	IAA (μg/mL)	
			0.1%	1.0%
JD-L31f	31.65 ± 1.90 a	17.69 ± 1.77 e	10.14 ± 0.95 e	36.72 ± 2.51 h
JD-R21f	15.89 ± 1.23 g	19.72 ± 2.40 cd	15.43 ± 1.47 d	40.91 ± 4.77 gh
CA-R11f	15.44 ± 1.29 g	17.57 ± 0.74 e	11.89 ± 1.05 e	39.84 ± 0.70 gh
TL-B11f	20.05 ± 0.32 e	21.74 ± 1.72 b	18.23 ± 3.30 d	77.42 ± 1.63 b
TL-B21f	28.96 ± 1.57 b	17.09 ± 1.52 e	50.23 ± 3.96 a	70.45 ± 2.09 c
TL-B31f	27.63 ± 1.10 bcd	24.41 ± 0.77 a	25.36 ± 1.91 c	95.29 ± 4.44 a
FY-G-R31f	26.86 ± 0.67 cd	21.87 ± 1.29 b	17.04 ± 1.77 d	96.50 ± 2.72 a
FY-R11f	26.19 ± 2.05 d	21.23 ± 1.34 bc	11.04 ± 2.14 e	59.57 ± 1.44 e
FY-R31f	11.91 ± 1.46 h	18.29 ± 0.62 de	17.32 ± 0.87 d	58.63 ± 0.83 e
FY-R41f	17.53 ± 1.38 f	17.37 ± 2.42 e	25.12 ± 1.83 c	64.95 ± 2.98 d
FY-R71f	28.43 ± 1.32 bc	21.06 ± 1.52 bc	9.82 ± 0.55 e	53.44 ± 1.12 f
LA-R21f	18.81 ± 0.80 ef	24.16 ± 0.62 a	16.27 ± 0.74 d	52.03 ± 1.08 f
YH-S11f	27.12 ± 1.09 cd	17.78 ± 1.19 e	15.76 ± 1.58 d	43.41 ± 2.76 g
YH-R21f	27.00 ± 1.69 cd	24.66 ± 1.39 a	31.18 ± 0.30 b	65.19 ± 5.07 d
YH-R31f	26.51 ± 1.05 d	23.66 ± 1.00 a	8.79 ± 1.45 e	38.51 ± 0.80 gh

Values that are separated by distinct lowercase letters within the same column indicate a significant difference at *p* < 0.05.

### 3.2 Effect of PGPF on rice growth

**Figure 3:**
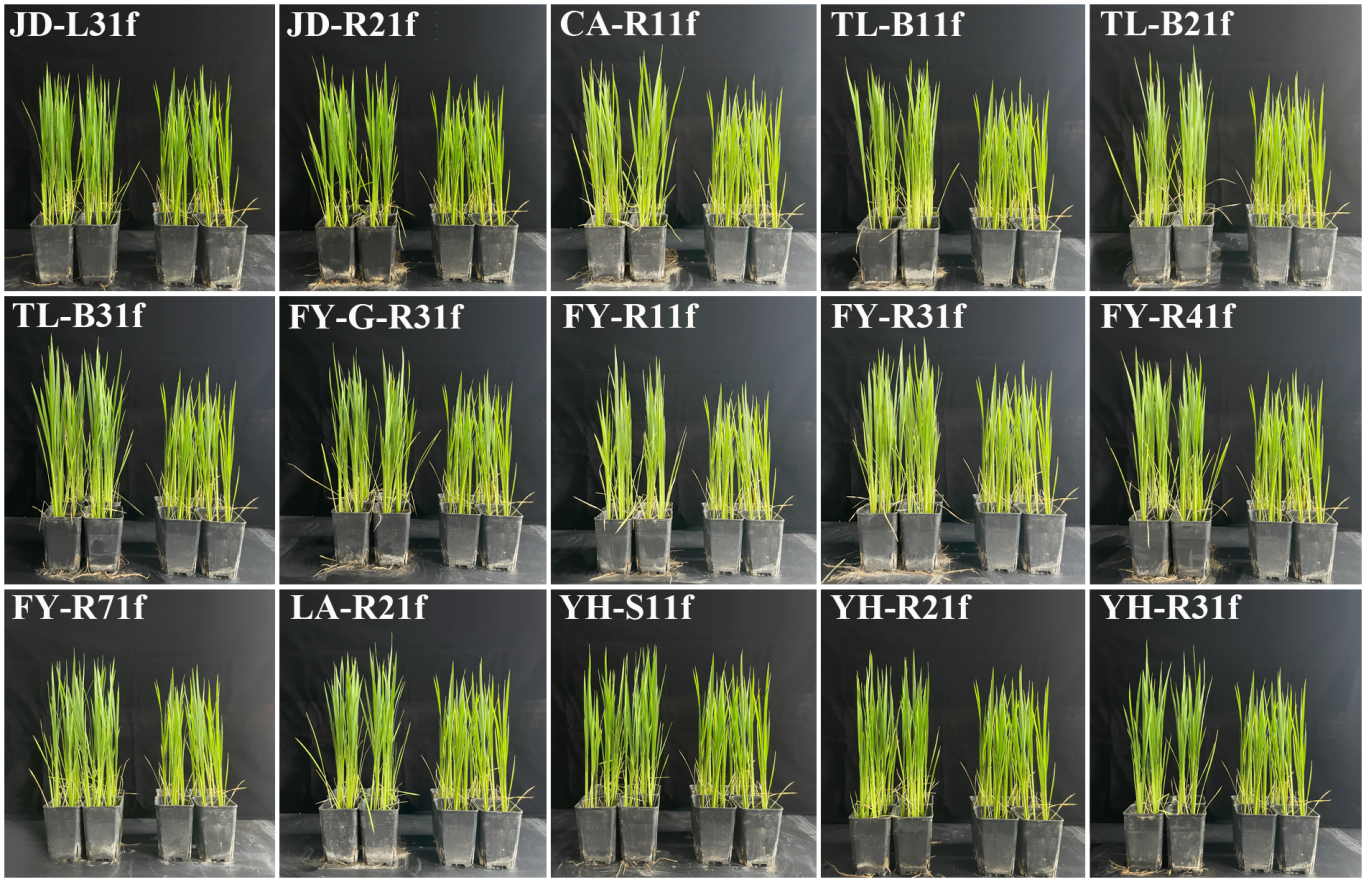
Effect of 15 fungal isolates on rice growth in a greenhouse, 55 days after inoculation.

All 15 fungal isolates were further examined for their growth-promoting activity in rice seedlings. After 55 days of inoculation, all 15 fungal isolates could cause a noticeable increase in rice growth compared to the control based on the phenotypic observation (**Figure 3**). The measured data further demonstrated that the fungal 15 isolates significantly (*p* < 0.05) affected the growth and biomass accumulation of rice seedlings compared to the control (**Table 2**). Indeed, among all treatments, rice seedlings inoculated with various fungal isolates exhibited different increases in plant height. Isolate TL-B31f exhibited the highest increase in plant height (359.59 mm), which was about 1.15 times that of the control. Isolates FY-R11f and FY-R31f followed, with 14.58% and 13.90% increases in plant height, respectively, as compared to that of control seedlings. Moreover, the fungal strains exhibited significantly (*p* < 0.05) different growth-promoting effects on root length of seedlings. Isolate FY-R41f showed the highest increase in root length (205.47 mm), with an increase of 43.31% compared to that of the control, followed by isolates FY-G-R31f and TL-B11f with an increase of 37.41% and 33.98% compared to that of the control, respectively. Furthermore, compared to the control, the fresh weights of seedlings were significantly (*p* < 0.05) increased by isolates CA-R11f, FY-R41f, and TL-B31f. In particular, the highest fresh weight of seedlings was achieved by isolate CA-R11f, with an increase of 95.09% with respect to that of the control. In addition, the dry weight of seedlings treated by isolates FY-R41f, FY-R31f, and FY-R11f was significantly (*p* < 0.05) higher than that of the control, with increases of 28.12%, 19.58%, and 16.39%, respectively. In general, the quality of rice seedlings treated with different fungal isolates was higher than that of the control.

**Table 2:** Effects of 15 fungal isolates on rice growth.

Treatments	PH (mm)	GPE%	RL (mm)	GPE%
JD-L31f	316.48 ± 8.08	1.48 g	158.83 ± 12.92	10.78 g
JD-R21f	344.11 ± 11.65	10.34 de	166.45 ± 12.63	16.09 f
CA-R11f	348.21 ± 12.37	11.65 cd	189.19 ± 9.71	31.95 c
TL-B11f	349.32 ± 13.88	12.01 cd	192.10 ± 8.58	33.98 bc
TL-B21f	346.09 ± 8.79	10.98 d	161.45 ± 12.55	12.60 fg
**TL-B31f**	**359.59 ± 12.49**	**15.30 a**	**191.57 ± 14.02**	**33.62 bc**
FY-G-R31f	346.04 ± 10.39	10.96 d	197.01 ± 10.68	37.41 b
FY-R11f	357.35 ± 11.68	14.58 ab	185.04 ± 10.35	29.06 cd
FY-R31f	355.21 ± 10.04	13.90 abc	185.44 ± 9.88	29.33 cd
**FY-R41f**	**355.02 ± 12.15**	**13.84 abc**	**205.47 ± 6.92**	**43.31 a**
FY-R71f	337.51 ± 13.36	8.22 e	159.38 ± 13.31	11.16 fg
LA-R21f	350.17 ± 6.05	12.28 bcd	184.60 ± 10.93	28.75 cd
YH-S11f	326.45 ± 9.88	4.68 f	155.75 ± 9.02	8.63 g
YH-R21f	324.82 ± 8.35	4.15 f	176.35 ± 10.60	23.00 e
YH-R31f	338.43 ± 12.78	8.52 e	179.71 ± 10.50	25.34 de
Control	311.86 ± 10.24	–	143.38 ± 8.52	–
Treatments	SFW (g)	GPE%	SDW (g)	GPE%
JD-L31f	1.14 ± 0.09	59.39 de	0.32 ± 0.04	-1.04 def
JD-R21f	1.04 ± 0.12	45.03 f	0.29 ± 0.04	-8.21 f
CA-R11f	1.40 ± 0.08	95.09 a	0.37 ± 0.06	15.22 abc
TL-B11f	1.24 ± 0.09	72.63 bc	0.34 ± 0.08	7.11 bcde
TL-B21f	1.25 ± 0.10	74.42 bc	0.35 ± 0.07	10.26 bcd
**TL-B31f**	**1.28 ± 0.15**	**78.58 b**	**0.35 ± 0.06**	**9.31 bcd**
FY-G-R31f	1.24 ± 0.18	72.09 bc	0.35 ± 0.08	8.66 bcde
FY-R11f	1.18 ± 0.17	63.93 cde	0.37 ± 0.07	16.39 abc
FY-R31f	1.26 ± 0.12	75.63 bc	0.38 ± 0.06	19.58 ab
**FY-R41f**	**1.36 ± 0.16**	**89.77 a**	**0.41 ± 0.09**	**28.12 a**
FY-R71f	1.09 ± 0.15	52.24 ef	0.33 ± 0.08	2.25 cdef
LA-R21f	1.22 ± 0.14	69.65 bcd	0.34 ± 0.05	5.92 bcde
YH-S11f	1.09 ± 0.08	51.97 ef	0.29 ± 0.03	-8.11 f
YH-R21f	0.87 ± 0.10	21.41 g	0.34 ± 0.04	5.38 bcdef
YH-R31f	1.11 ± 0.11	54.66 ef	0.30 ± 0.05	-5.12 ef
Control	0.72 ± 0.09	–	0.32 ± 0.05	–

PH, plant height; RL, root length; SFW, seedling fresh weight; SDW, seedling dry weight; GPE, growth promotion efficacy. Means are averages ± SD. Different lowercase letters within the same columns reveal the significance among different treatments (*p* < 0.05). The two treatments with the best growth-promoting properties are shown in bold.

### 3.3 Fungal identification

**Figure 4:**
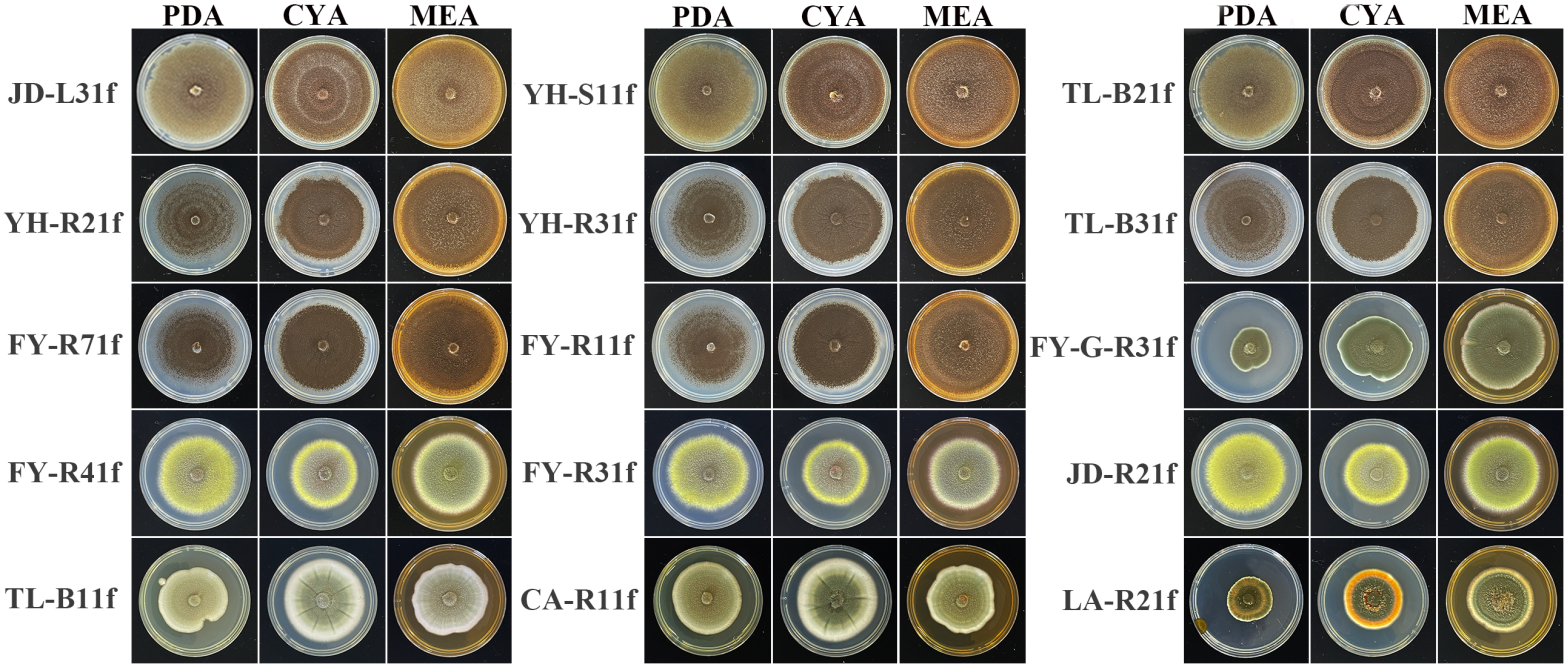
Colony features on PDA (potato dextrose agar), CYA (czapek yeast autolysate agar), and MEA (malt extract agar) after 7 days at 25°C.

After being cultured on PDA, CYA, and MEA medium at 25°C for 7 days, it was revealed that 15 fungal isolates belong to *Aspergillus* sp., *Penicillium* sp., and *Talaromyces* sp. based on the obvious distinctions of colony morphology (**Figure 4**). Indeed, eight isolates including JD-L31f, YH-S11f, TL-B21f, YH-R21f, YH-R31f, TL-B31f, FY-R71f, and FY-R11f were attributed to the genus *Aspergillus* due to the coffee brown to black colonies. Three isolates including FY-G-R31f, TL-B11f, and CA-R11f were attributed to the genus *Penicillium* in view of the irregular, green, velvety texture of colonies. Four isolates including LA-R21f, FY-R41f, FY-R31f, and JD-R21f were attributed to the genus *Talaromyces* due to the yellow-green colonies and a characteristic red pigment (Tsang et al., 2018; Houbraken et al., 2020). To further identify the species of all isolates, phylogenetic analysis was conducted by using the maximum likelihood method based on sequences of several conserved genes including ITS, TUB, CaM, and RPB2 of the tested isolates and closely related species (**Table 3**). The phylogenetic trees with the highest log likelihood were drawn, with branch lengths reflecting evolutionary distance and bootstrap proportions beside the branches indicating credibility.

**Table 3:** Fungal isolates used in this study.

Species	Isolates	GenBank accession number			
		ITS	TUB	CaM	RPB2
*Talaromyces aculeatus*	NRRL2129	KF741995	KF741929	KF741975	MH793099
*T. apiculatus*	CBS312.59	JN899375	JX091378	KF741950	KM023287
*T. aurantiacus*	CBS314.59	JN899380	KF741917	KF741951	KX961285
*T. derxii*	CBS412.89	JN899327	JX494306	KF741959	KM023282
*T. flavovirens*	CBS102801	JN899392	JX091376	KF741933	KX961283
*T. flavus*	CBS310.38	MH867464	JX494302	KF741949	JF417426
*T. helicus*	CBS335.48	MH856373	KJ865725	KJ885289	KM023273
*T. muroii*	CBS756.96	MK450747	KJ865727	KJ885274	KX961276
*T. pinophilu*	NRRL62183	MH909497	MH909388	MH909444	MH909550
***T. purpureogenus***	B195_0419	OR192900	OR233627	OR327660	OR211408
	**LA-R21f**	**PQ269783**	**PQ516235**	**PQ516220**	**PQ516268**
*T. ruber*	CBS195.88	JX965240	JX965350	JX965204	JX965310
*T. rubicundus*	CBS342.59	JN899384	JX494309	KF741956	KM023296
*T. sayulitensis*	NRRL62272	MH793083	MH792956	MH793020	MH793147
*T. stollii*	CBS624.93	JX315676	JX315636	JX315644	JX965315
***T. veerkampii***	NRRL 6095	MH793040	MH792912	MH792976	MH793103
	**FY-R31f**	**PQ269785**	**PQ516237**	**PQ516222**	**PQ516270**
	**FY-R41f**	**PQ269784**	**PQ516236**	**PQ516221**	**PQ516269**
	**JD-R21f**	**PQ269786**	**PQ516238**	**PQ516223**	**PQ516271**
*T. viridulus*	CBS 252.87	JN899314	JX091385	KF741943	JF417422
*Aspergillus aculeatus*	CBS172.66	FJ629320	HE577806	FN594542	JN121448
*A. aculeatinus*	CBS121060	MH863102	EU159220	EU159241	HF559233
*A. amoenus*	NRRL4838	EF652480	JN853946	JN854035	JN853824
*A. austroafricanus*	NRRL233	JQ301891	JN853963	JN854025	JN853814
*A. baeticus*	CCF4226	HE615086	HE615092	HE615117	HE615124
*A. brevijanus*	NRRL1935	EF669582	EU014078	EF669540	EF669624
***A. brunneoviolaceus***	NRRL4912	EF661220	EF661105	EF661147	EF661045
	**JD-L31f**	**PQ269772**	**PQ516224**	**PQ516209**	**PQ516257**
	**YH-S11f**	**PQ269773**	**PQ516225**	**PQ516210**	**PQ516258**
	**TL-B21f**	**PQ269774**	**PQ516226**	**PQ516211**	**PQ516259**
*A. campestris*	NRRL13001	EF669577	EU014091	EF669535	EF669619
*A. candidus*	NRRL303	EF669592	EU014089	EF669550	EF669634
*A. capensis*	DTO179E6	KJ775550	KJ775072	KJ775279	KP987020
*A. creber*	NRRL58592	JQ301889	JN853980	JN854043	JN853832
*A. cvjetkovicii*	NRRL227	EF652440	EF652264	EF652352	EF652176
*A. flavipes*	NRRL302	EF669591	EU014085	EF669549	EF669633
*A. fructus*	NRRL239	EF652449	EF652273	EF652361	EF652185
*A.iizukaelo*	NRRL3750	EF669597	EU014086	EF669555	EF669639
*A. janus*	NRRL1787	EF669578	EU014076	EF669536	EF669620
*A. jensenii*	NRRL58600	JQ301892	JN854007	JN854046	JN853835.
*A. protuberus*	NRRL3505	EF652460	EF652284	EF652372	EF652196
*A. puniceus*	NRRL5077	EF652498	EF652322	EF652410	EF652234
*A. puulaauensis*	NRRL35641	JQ301893	JN853979	JN854034	JN853823
*A. saccharolyticus*	CBS127449	HM853552	HM853553	HM853554	HF559235
*A. subalbidus*	NRRL5214	LT908114	LT908034	LT908035	LT908036
*A. subversicolor*	NRRL58999	JQ301894	JN853970	JN854010	JN853799
*A. sydowii*	NRRL250	EF652450	EF652274	EF652362	EF652186
*A. tabacinus*	NRRL4791	EF652478	EF652302	EF652390	EF652214
*A. tennesseensis*	NRRL13150	JQ301895	JN853976	JN854017	JN853806
*A. tritici*	CBS266.81	MN431381	MN969368	MN969233	MN969098
***A. tubingensis***	NRRL62644	KC796398	KC796370	KC796386	KC796437
	**YH-R21f**	**PQ269775**	**PQ516227**	**PQ516212**	**PQ516260**
	**YH-R31f**	**PQ269776**	**PQ516228**	**PQ516213**	**PQ516261**
	**TL-B31f**	**PQ269777**	**PQ516229**	**PQ516214**	**PQ516262**
	**FY-R71f**	**PQ269778**	**PQ516230**	**PQ516215**	**PQ516263**
	**FY-R11f**	**PQ269779**	**PQ516231**	**PQ516216**	**PQ516264**
*A. ustus*	NRRL4991	EF652492	EF652316	EF652404	EF652228
*A. venenatus*	NRRL13147	JQ301896	JN854003	JN854014	JN853803
*A. versicolor*	NRRL238	EF652442	EF652266	EF652354	EF652178
*P. alfredii*	DTO269A4	KJ775684	KJ775177	KJ775411	KJ834520
*P. brefeldianum*	NRRL710	AF033435	EU021669	EU021683	EU021658
*P. incoloratum*	CBS101753	KJ834508	KJ834457	KJ866984	JN406651
*P. jamesonlandense*	IBT21985	KY989164	KY989039	KY989101	KY989214
*P. janthinellum*	CBS340.48	GU981585	GU981625	MN969268	GU981625
*P. javanicum*	CBS341.48	GU981613	GU981657	MN969269	JN121498
***P. limosum***	CBS339.97	NR_111496	GU981621	MN969271	KF296433
	**TL-B11f**	**PQ269780**	**PQ516232**	**PQ516217**	**PQ516265**
	**CA-R11f**	**PQ269781**	**PQ516233**	**PQ516218**	**PQ516266**
*P. malacaense*	NRRL35754	EU427300	EU427268	KF932944	EU427261
*P. nodulum*	CBS227.89	KC411703	KJ834475	KJ867003	JN406603
***P. oxalicum***	CBS219.30	MH855125	KF296462	MN969283	JN121456
	**FY-G-R31f**	**PQ269782**	**PQ516234**	**PQ516219**	**PQ516267**
*P. penarojense*	CBS113178	GU981570	GU981646	MN969287	KF296450
*P. piscarium*	CBS362.48	GU981600	GU981668	MN969288	KF296451
*P. raistrickii*	CBS261.33	JN617697	KJ834485	KJ867006	JN606589
*P. ribeum*	CBS127809	MH864716	MN969395	KJ866995	JN406631
*P. sajarovii*	CBS277.83	KC411724	MN969397	KJ867007	JN406588
*P. soppii*	CBS226.28	MH854995	MN969399	KJ867002	JN406606
*P. vanderhammenii*	CBS126216	MH863982	GU981647	MN969308	KF296458
*P. virgatum*	CBS114838	AJ748692	KJ834500	KJ866992	JN406641
*P. wotroi*	CBS118171	GU981591	GU981637	MN969313	KF296460
*P. zonatum*	CBS992.72	GU981581	GU981651	MN969315	KF296461

Species and sequences obtained from this study are shown in bold.

**Figure 5:**
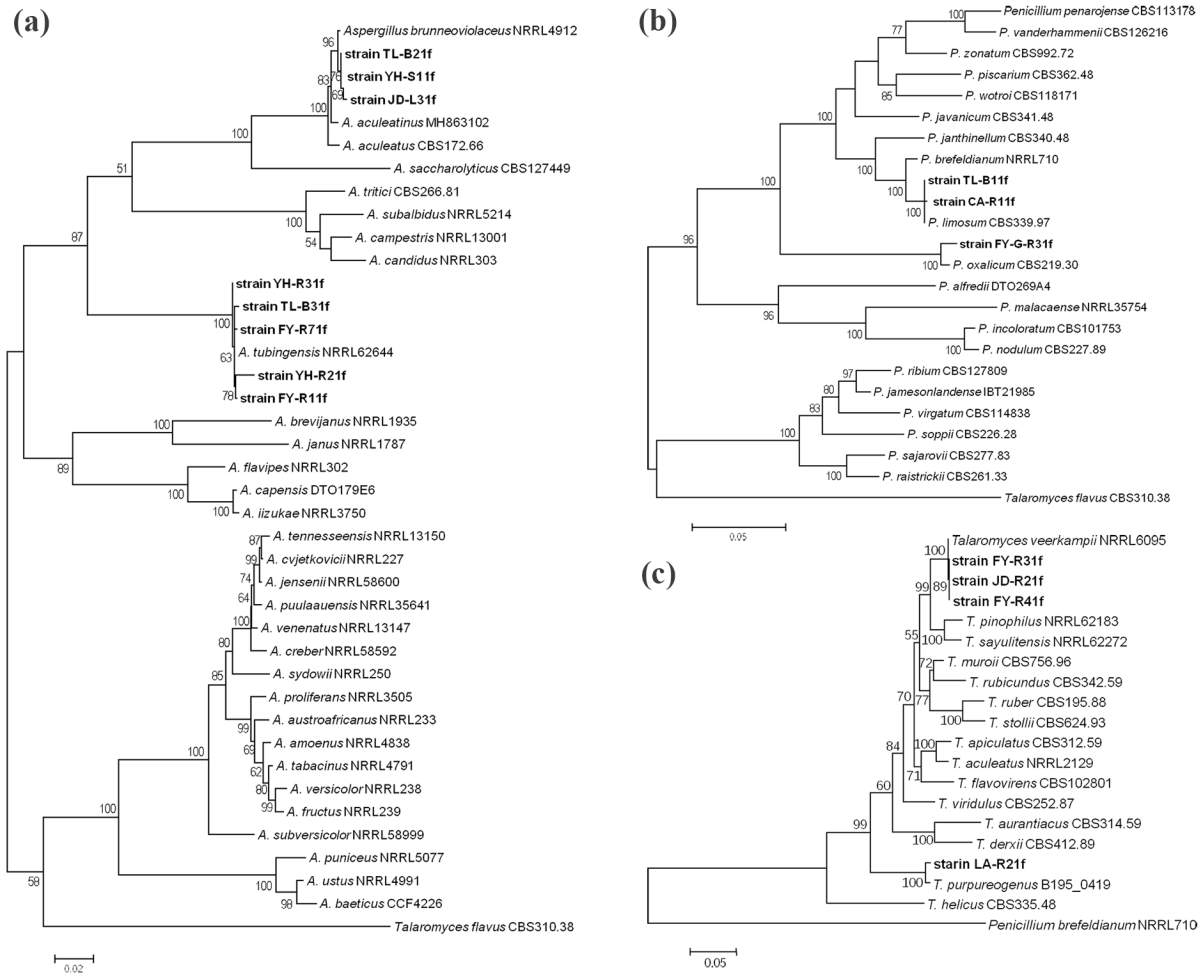
Maximum likelihood (ML) tree generated with MEGA 7.0 from the combined ITS, TUB, CaM, and RPB2 gene sequences of *Aspergillus* **(a)**, *Penicillium* **(b)**, and *Talaromyces* **(c)**, respectively. The reference sequences of closely related species were gotten from NCBI, and the isolates obtained from soil in this study were shown in bold letters. Bootstrap values after 1000 replicates were expressed as percentages.

The phylogenetic trees showed that all 15 isolates were separated into six distant clades (**Figure 5**). Indeed, isolates JD-L31f, YH-S11f, and TL-B21f were clustered with *A. brunneoviolaceus* isolate NRRL4912 as a distinct clade with a bootstrap of 96%, suggesting the three isolates should be considered as *A. brunneoviolaceus*. Isolates YH-R21f, YH-R31f, TL-B31f, FY-R71f, and FY-R11f were clustered with *A. tubingensis* isolate NRRL62644 as a distinct clade with a bootstrap of 100%, suggesting the five isolates should be considered as *A. tubingensis* (**Figure 5a**). Isolate FY-G-R31f was clustered with *Penicillium oxalicum* isolate CBS219.30 as a distinct clade with a bootstrap of 100%, suggesting this isolate should be considered as *P. oxalicum*. Isolates TL-B11f and CA-R11f were clustered with *Penicillium limosum* isolate CBS339.97 as a distinct clade with a bootstrap of 100%, suggesting the two isolates should be considered as *P. limosum* (**Figure 5b**). Isolate LA-R21f clustered with *Talaromyces purpureogenus* isolate B195_0419 as a distinct clade with a bootstrap of 100%, suggesting this isolate should be considered as *T. purpureogenus*. Isolates FY-R41f, FY-R31f, and JD-R21f were clustered with *T. veerkampii* isolate NRRL6095 as a distinct clade with a bootstrap of 100%, suggesting the three isolates should be considered as *T. veerkampii* (**Figure 5c**).

### 3.4 Improvement of soil quality, microbial community structure, and function by PGPF

Two PGPF isolates TL-B31f and FY-R41f, with the best growth-promoting properties, were selected for analyzing their effects on soil quality, microbial community composition, and function after 55 days of inoculation. As shown in **Table 4**, compared with the control, the soil pH and TP were generally unaffected by At-TLB31f (inoculated with isolate TL-B31f) and Tv-FYR41f (inoculated with isolate FY-R41f) treatments. Meanwhile, At-TLB31f treatment caused a 4.38% decrease (*p* < 0.05) in SOM compared to the control, while there was no significant difference between Tv-FYR41f treatment and the control. Furthermore, AP was significantly (*p* < 0.05) increased by At-TLB31f (42.52%) and Tv-FYR41f (48.51%) treatments compared to the control.

**Table 4:** Impacts of two different PGPF isolates on soil quality properties.

Treatments	pH	SOM (g/kg)	TP (g/kg)	AP (mg/kg)
At-TLB31f	8.00 ± 0.05 ab	8.55 ± 0.15 b	0.88 ± 0.00 a	27.33 ± 0.71 b
Tv-FYR41f	8.06 ± 0.03 a	9.01 ± 0.12 a	0.88 ± 0.01 a	28.47 ± 0.32 a
Control	7.97 ± 0.05 b	8.94 ± 0.29 a	0.87 ± 0.01 a	19.17 ± 0.63 c

Values that are separated by distinct lowercase letters within the same column indicate a significant difference at *p* < 0.05. SOM is soil organic matter; TP is total phosphorus; AP is available phosphorus. At-TLB31f, treatment inoculated with treatment inoculated with isolate TL-B31f; Tv-FYR41f, treatment inoculated with treatment inoculated with isolate FY-R41f.

**Figure 6:**
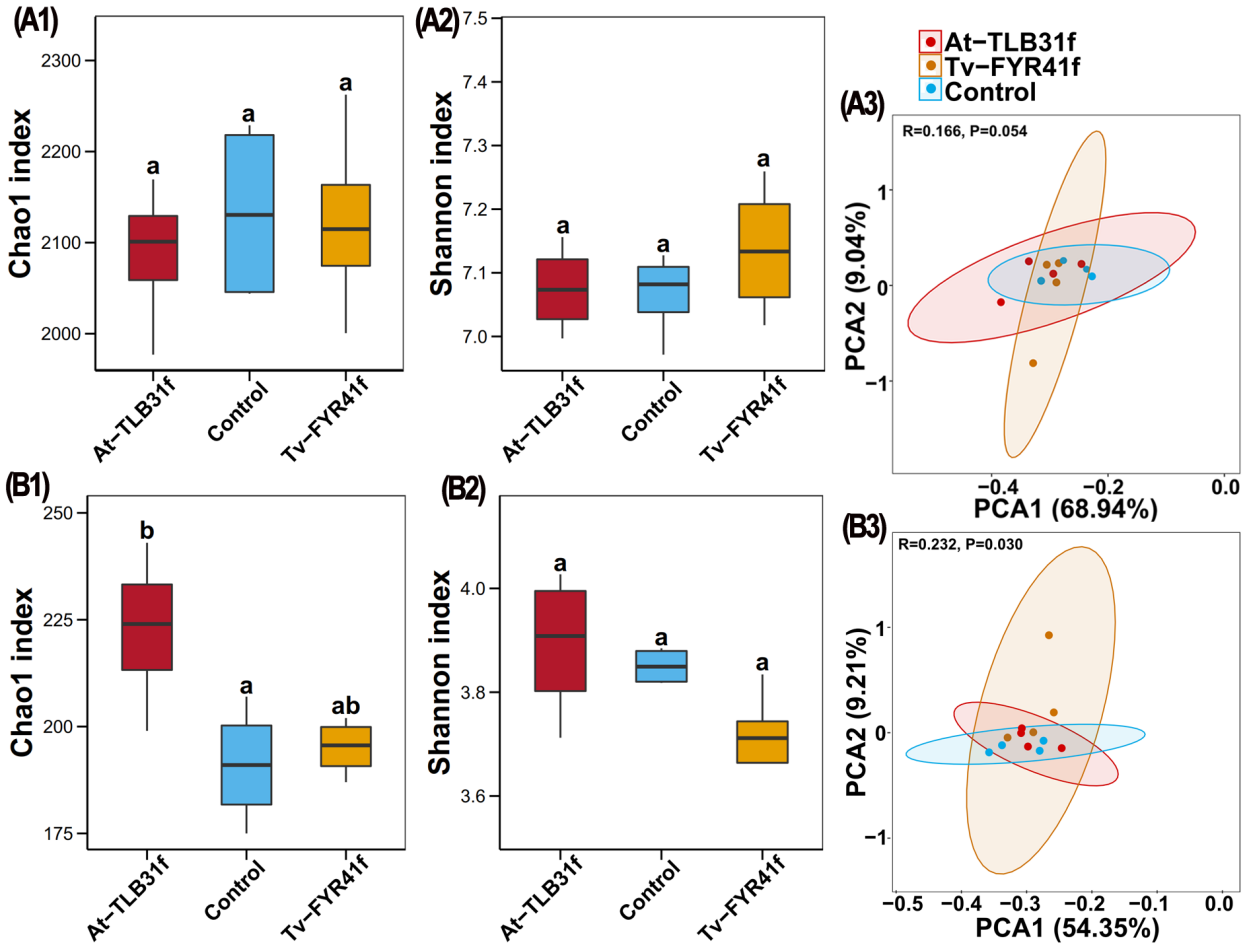
The effect of two different PGPF isolates on the Chao1 and Shannon index of bacteria **(A1, A2)** and fungi **(B1, B2)**. Different lowercase letters reveal the significance among different treatments (*p* < 0.05). Principal component analysis (PCA) results of soil bacterial **(A3)** and fungal **(B3)** communities based on OTUs abundance. Ellipses have been drawn for each treatment with a confidence limit of 0.95.

Based on high-throughput amplicon sequencing of DNA extracted from soil samples, bacterial and fungal taxonomic diversity (*α*-diversity) and composition (*β*-diversity) were compared between treatments inoculated by isolate TL-B31f or FY-R41f and the control. Indeed, the average bacterial Chao1 index was 2,087 (1,977–2,169), 2,123 (2,000–2,262), 2,133 (2,043–2,228), and the average bacterial Shannon index was 7.07 (7.00–7.16), 7.14 (7.02–7.26), and 7.07 (6.97–7.13) in At-TLB31f, Tv-FYR41f treatments, and the control, respectively (**Figures 6A1, A2**). In general, the bacterial Chao1 index was lower (2.17% and 0.48%) in At-TLB31f and Tv-FYR41f treatments, along with a higher Shannon index (0.13% and 1.00%) than that in the control. Meanwhile, the average fungal Chao1 index was 223 (199–243), 195 (187–202), 191 (175–207), and the average fungal Shannon index was 3.89 (3.71–4.03), 3.70 (3.53–3.83), and 3.85 (3.82–3.88) in At-TLB31f, Tv-FYR41f treatments, and the control, respectively (**Figures 6B1, B2**). In other words, isolate TL-B31f inoculation caused a 16.49% (*p* < 0.05) and 1.00% increase in the fungal Chao1 and Shannon index of soil, while isolate FY-R41f inoculation caused a 2.21% increase and 4.00% reduction, respectively, compared to the control. Overall, there was no significant (*p* < 0.05) difference in the *α*-diversity of soil bacteria between two PGPF isolates treatments and the control, but the *α*-diversity of soil fungi was significantly (*p* < 0.05) changed by inoculation of isolates TL-B31f and FY-R41f.

Results of PCA revealed that the OTU abundance from 12 soil samples of At-TLB31f, Tv-FYR41f treatments, and the control comprised three different groups, but there was noticeable overlap among all three different treatments, regardless of bacterial or fungal data (**Figures 6A3, B3**). Indeed, the two principal components accounted for 77.98% (PC1 68.94%, PC2 9.04%, respectively) and 63.56% (PC1 54.35%, PC2 9.21%, respectively) of the total variance in the bacterial and fungal communities, respectively. PERMANOVA performed on all samples also indicated that different PGPF explained 16.6% (*p* = 0.054) and 23.2% (*p* = 0.030) of the variation, respectively. These findings showed that the bacterial and fungal communities in soil inoculated by isolate TL-B31f or FY-R41f were generally similar to the control, albeit with some differences.

**Figure 7:**
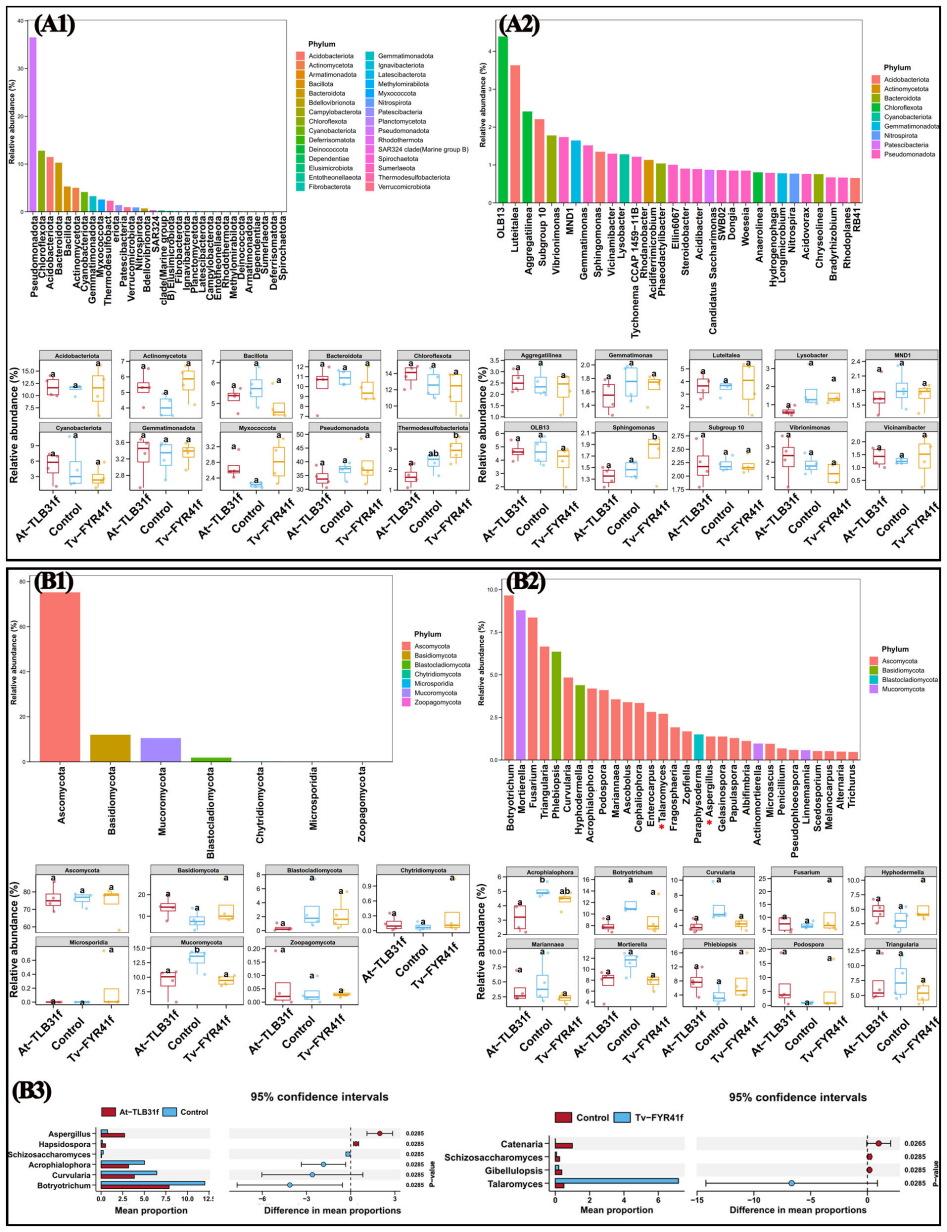
Relative abundance of dominant bacteria **(A)** and fungi **(B)** at the phylum **(A1, B1)** and genus **(A2, B2)** level. The abundance of fungal biomarkers at the genus level between At-TLB31f (or Tv-FYR41f) treatments and the control **(B3)**.

The compositional differences of soil bacterial and fungal communities among the three different treatments were compared at the phylum and genus levels. Indeed, in the bacterial community, Pseudomonadota, Chloroflexota, Acidobacteriota, Bacteroidota, Bacillota, Actinomycetota, Cyanobacteriota, Gemmatimonadota, Myxococcota, and Thermodesulfobact were the top 10 dominant phyla (**Figure 7A1**), and *OLB13*, *Luteitalea*, *Aggregatilinea*, *Subgroup 10*, *Vibrionimonas*, *MND1*, *Gemmatimonas*, *Sphingomonas*, *Vicinamibacter*, and *Lysobacter* were the top 10 dominant genera (**Figure 7A2**). Compared with the control, the RAs of *Vibrionimonas* (22.12%) and *Vicinamibacter* (10.13%) were increased, while *Lysobacter* (62.56%), *Gemmatimonas* (11.76%), *Sphingomonas* (8.17%, *p* < 0.05), and *MND1* (7.64%) were decreased in At-TLB31f treatment; the RAs of *Vibrionimonas* (30.15%), *OLB13* (17.32%), *Aggregatilinea* (11.83%), *Lysobacter* (10.29%), and *MND1* (6.79%) were decreased, while *Sphingomonas* (19.68%, *p* < 0.05), *Vicinamibacter* (7.92%), and *Luteitalea* (5.73%) were increased in Tv-FYR41f treatment (**Figure 7A2**).

Similarity, according to the distribution and RAs of fungi among all three different treatments at the phylum level, Ascomycota, Basidiomycota, Mucoromycota, and Blastocladiomycota were the main fungal phyla, with abundance of 73.80–76.11%, 8.12–14.09%, 9.20–13.03%, and 0.40–2.93%, respectively (**Figure 7B1**). Furthermore, at the genus level, *Botryotrichum*, *Mortierella*, *Fusarium*, *Triangularia*, *Phlebiopsis*, *Curvularia*, *Hyphodermella*, *Acrophialophora*, *Podospora*, and *Mariannaea* were the top 10 dominant genera (**Figure 7B2**). Compared with the control, the At-TLB31f treatment caused a 40.29%, 36.68%, 34.39%, 33.16%, 24.37%, and 9.45% reduction in *Curvularia*, *Acrophialophora* (*p* < 0.05), *Botryotrichum*, *Mortierella*, *Mariannaea*, and *Triangularia*, respectively, while a 641.74%, 98.87%, 49.74%, and 6.07% increase in *Podospora*, *Phlebiopsis*, *Hyphodermella*, and *Fusarium*, respectively (**Figure 7B2**). In addition, the RAs of *Aspergillus* were significantly increased by 264.68% in At-TLB31f treatment, while *Talaromyces* were significantly increased by 1328.35% in Tv-FYR41f treatment (**Figure 7B3**). In other words, compared to the control, the microbial community composition of the two PGPF isolates treatments was reconstructed with an increase or decrease of some specific microbes.

**Figure 8:**
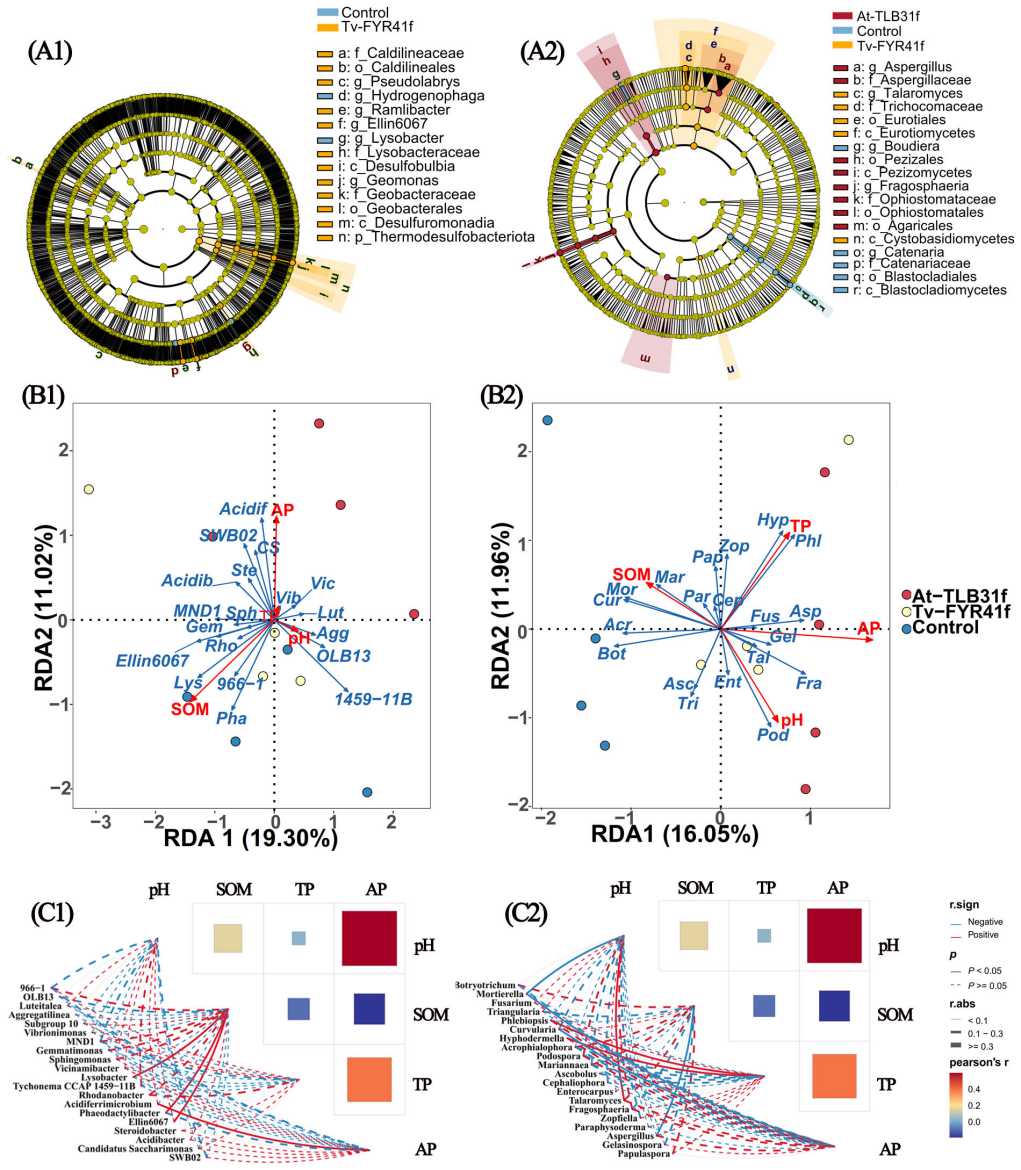
Liner discriminant analysis (LDA) effect size evaluation of bacterial **(A1)** or fungal **(A2)** taxa revealed the most differentially abundant taxa among At-TLB31f, Tv-FYR41f treatments, and the control soil communities. Only bacterial taxa with LDA > 3 (*p* < 0.05) and fungal taxa with LDA > 3.5 (*p* < 0.05) were shown. RDA (redundancy discriminant analysis) comparing soil characteristics to bacterial **(B1)** or fungal **(B2)** populations at the genus level. Diagrams of correlation network between soil properties and bacterial taxa **(C1)** or fungal taxa **(C2)** among three different treatments. *Lut, Luteitalea, Agg, Aggregatilinea, Vib, Vibrionimonas, Gem, Gemmatimonas, Sph, Sphingomonas, Vic, Vicinamibacter, Lys, Lysobacter, 1459-11B, Tychonema CCAP 1459-11B, Rho, Rhodanobacter, Acidif, Acidiferrimicrobium, Pha, Phaeodactylibacter, Ste, Steroidobacter, Acidib, Acidibacter, CS, Candidatus Saccharimonas*, *Bot, Botryotrichum, Mor, Mortierella, Fus, Fusarium, Tri, Triangularia, Phl, Phlebiopsis, Cur, Curvularia, Hyp, Hyphodermella, Acr, Acrophialophora, Pod, Podospora, Mar, Mariannaea, Asc, Ascobolus, Cep, Cephaliophora, Ent, Enterocarpus, Tal, Talaromyces, Fra, Fragosphaeria, Zop, Zopfiella, Par, Paraphysoderma, Asp, Aspergillus, Gel, Gelasinospora, Pap, Papulaspora*. SOM, soil organic matter; TP, total phosphorus; AP, available phosphorus.

Furthermore, LEfSe was carried out to identify specific bacterial and fungal biomarkers distinguishing soil microbial communities among At-TLB31f, Tv-FYR41f treatments, and the control (**Figure 8A**). Results showed that 14 bacterial biomarkers (LDA > 3.0) and 18 fungal biomarkers (LDA > 3.5) were present in all treatments. In fact, communities of At-TLB31f treatment were enriched with Agaricales, *Aspergillus*, Aspergillaceae, *Fragosphaeria*, Pezizales, Pezizomycetes, Ophiostomataceae, and Ophiostomatales. Communities of Tv-FYR41f treatment were enriched with Caldilineaceae, Caldilineales, Desulfobulbia, Desulfuromonadia, *Ellin6067*, Geobacteraceae, Geobacterales, *Geomonas*, Lysobacteraceae, *Pseudolabrys*, *Ramlibacter*, Thermodesulfobacteriota, and Cystobasidiomycetes, Eurotiales, Eurotiomycetes, Trichocomaceae, *Talaromyces*. The controls were enriched with *Hydrogenophaga*, *Lysobacter*, and Blastocladiomycetes, Blastocladiales, *Boudiera*, Catenariaceae, and *Catenaria*. Overall, these microbial taxa might play important roles in modulating rice growth.

RDA was performed to analyze how soil microbial community composition correlated with soil environmental factors. Indeed, SOM (*r^2^* = 0.74, *p* = 0.004) and AP *(r^2^* = 0.38, *p* = 0.117) were the most important factors in explaining the variation in the composition of the bacterial community, with axes 1 (19.30%) and axes 2 (11.02%) accounting for 30.32% of total variations (**Figure 8B1**). Meanwhile, AP (*r^2^* = 0.80, *p* = 0.004), TP (*r^2^* = 0.41, *p* = 0.080), pH (*r^2^* = 0.37, *p* = 0.135), and SOM (*r^2^* = 0.26, *p* = 0.281) were vital factors in explaining the variation in the composition of the fungal community, with axes 1 (16.05%) and axes 2 (11.96%) accounting for 28.01% of total variations (**Figure 8B2**). Simultaneously, correlation network analysis also indicated that soil properties obviously influence the composition of microbial communities at the genus level (**Figure 8C**). Results showed that *Ellin6067*, *Lysobacter*, and *Phaeodactylibacter* were significantly (*p* < 0.05) positively correlated with SOM. *Acidiferrimicrobium* was significantly (*p* < 0.05) positively correlated with AP, while *Curvularia* was significantly (*p* < 0.05) negatively correlated with AP (**Figure 8C1**). *Mortierella* was significantly (*p* < 0.05) negatively correlated with pH and AP. *Phlebiopsis* and *Hyphodermella* were significantly (*p* < 0.05) positively correlated with TP. *Talaromyces* was significantly (*p* < 0.05) positively correlated with pH, while *Aspergillus* was significantly (*p* < 0.05) negatively correlated with pH (**Figure 8C2**).

## 4 Discussion

Food security is a critical global issue that affects both the economy and livelihood, constituting a fundamental pillar of national food security (Liu et al., 2021). The foundation of food production greatly depends on the availability of quality and quantity of arable land. Unregulated expansion of non-grain crops not only jeopardizes food security by leading to shortages and imbalances in food supply but also results in the degradation of soil quality and environmental issues due to changes in land use practices (Cheng et al., 2022; Jiang et al., 2024). The artificial introduction of PGPF into soils represents a cost-effective and eco-friendly strategy for improving soil nutrient conditions and promoting crop growth (Hassan, 2017; Murali et al., 2021).

### 4.1 Isolation, identification and characterization of PGPF

**Figure 9:**
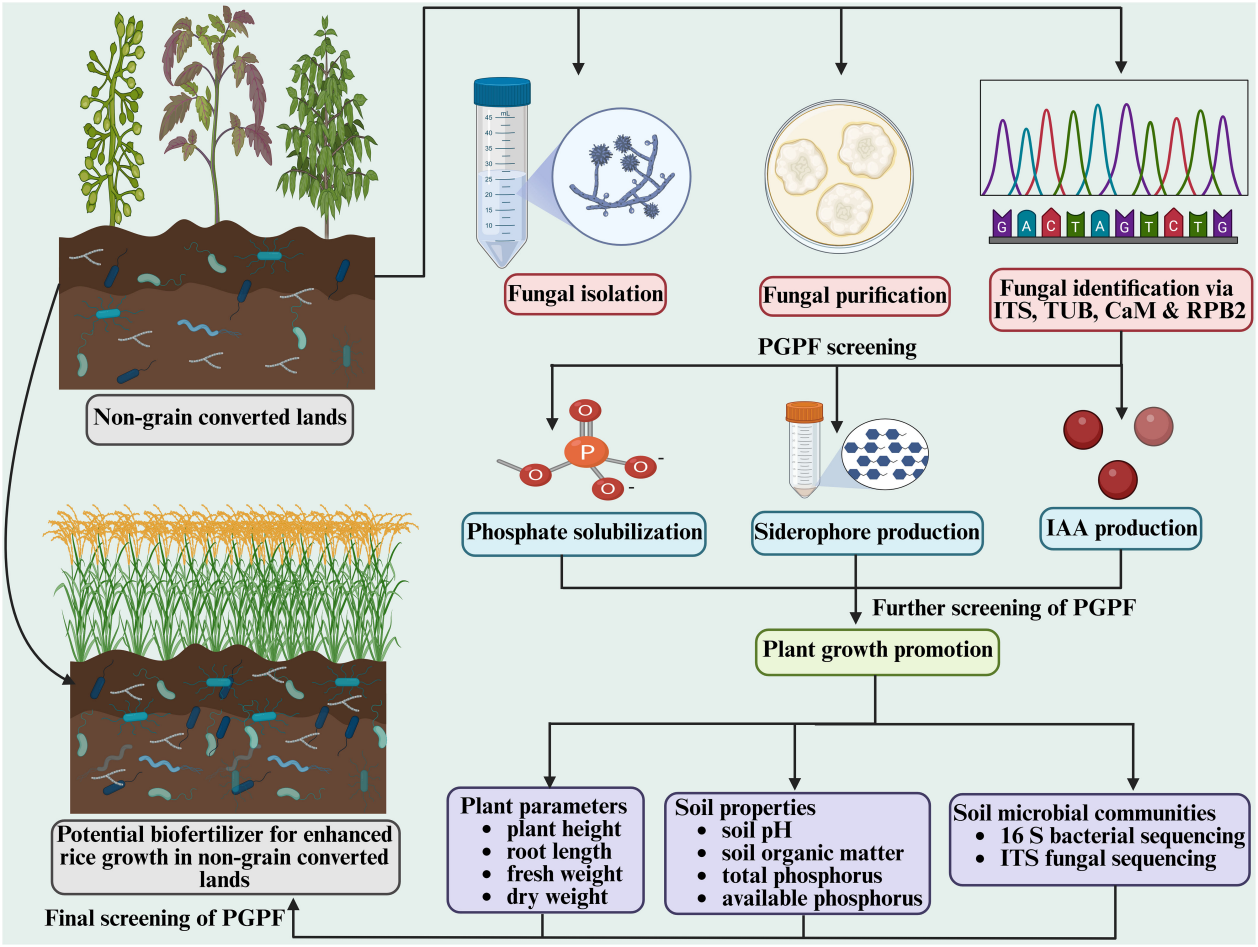
Schematic illustration of isolation, identification and characterization of multiple plant growth-promoting fungi (PGPF) and their effect on rice growth improvement on non-grain converted land.

In order to further enhance the productivity of land converted from non-grain to grain cultivation and facilitate the growth of food crops on such soils, we conducted an isolation of soil fungi by collecting soil samples from six sites where land use had been converted from non-grain to grain cultivation, yielding a total of 215 fungal isolates from 108 soil samples. These obtained fungal isolates were further screened and characterized based on their ability to solubilize phosphate, to produce siderophores, and to synthesize IAA, which has been widely reported to play an important role in growth promotion of plants (**Figure 9**).

Phosphorus is a key component of many vital substances and structures within plant cells, such as nucleoproteins, phospholipids, and nucleic acids. It plays a critical role in the physiological and biochemical processes throughout the plant’s life cycle and is indispensable for plant growth and development (Han et al., 2022). However, most phosphorus in the soil exists in insoluble inorganic or organic forms, making it difficult for plants to absorb and utilize (Wang et al., 2021). In this study, initial screening showed that 15 fungal isolates were capable of producing clear phosphate solubilization zones on NBRIP medium, demonstrating a broad ability to solubilize phosphate. Phosphate-solubilizing PGPF can dissolve insoluble forms of phosphorus or mineralize organic phosphorus through mechanisms such as the secretion of organic acids, excretion of protons, or enzymatic production, thereby making phosphorus available for plant uptake (Chen et al., 2019). In agreement with the result of this study, some fungal species including *A. brunneoviolaceus*, *Aspergillus niger*, *Nigrospora* sp*haerica*, *Penicillium corylophilum*, and *Penicillium chrysogenum* have been reported to enhance plant growth by participating in phosphate solubilization (Hassan, 2017; Li et al., 2023).

Additionally, previous studies showed that PGPF that produce siderophores also play a significant role in plant growth and development by improving plant resilience to stress and iron limitation (Sharma et al., 2024). Similarly, our study confirmed that all 15 fungal isolates possess the ability to produce siderophores. PGPF not only promotes crop growth by producing siderophores but also enhances the iron content of food crops, hence addressing nutritional deficiencies in human diets (Kaur et al., 2020). In addition, various phytohormones (particularly IAA) could be produced from PGPF, which could either directly enhance plant growth through regulating root growth, or indirectly induce tolerance to stress situation by modifying growth inhibitors (Ansabayeva et al., 2025). For instance, fungi-produced IAA has been reported to positively influence the root development of eggplant plants (Li et al., 2021). In agreement with previous results, our study indicated that IAA can be produced by the selected PGP fungal isolates in the presence of 0.1% and 1.0% tryptophan, indicating that the fungi isolated from the converted farmland have the potential to stimulate root growth and development. However, the IAA production of these fungal isolates can be affected by the concentration of tryptophan.

Interestingly, the bioassays indicated that all 15 fungal isolates screened in this study differed in their performance in promoting rice growth. For instance, isolates FY-R41f and FY-R31f did not display particularly strong capabilities in phosphate solubilization, siderophore production, or IAA synthesis during *in vitro* assays, yet they significantly enhanced rice seedling growth in bioassays, substantially increasing seedling height, root length, fresh and dry weight. The discrepancies between *in vitro* and *in vivo* results may be mainly due to differences in fungal metabolites or colonization abilities of fungal species in soil. Additionally, several plant growth-promoting isolates, including TL-B31f, FY-R11f, TL-B11f, and CA-R11f were non-pathogenic to the host rice, but significantly increased rice seedling height, root length, fresh weight, and dry weight. The PGP effect on rice seedlings depends on fungal isolates.

Based on a combination of morphological characteristics and phylogenetic analysis of multiple fungal gene sequences, all 15 fungal isolates were classified into six species: *A. brunneoviolaceus*, *A. tubingensis*, *P. oxalicum*, *P. limosum*, *T. veerkampii*, and *T. purpureogenus*. Previous studies have highlighted the potential applications of several of these isolates in agriculture through various mechanisms (**Table 5**). For example, *P. oxalicum* isolate UOM PGPF 16 had been used as PGPF to enhance plant growth and induce resistance in pearl millet against downy mildew disease (Murali and Amruthesh, 2015). *P. limosum* AK-7, isolated from soil, has demonstrated significant antifungal activity, making it a promising resource for developing biological pesticides (Basavarajappa et al., 2023). *T. purpureogenus* isolate M13026-2, isolated from mushroom substrate, could colonize in the rhizosphere soil stably, and significantly improve the growth of cucumber seedlings (Zhao et al., 2021). *A. tubingensis* demonstrated the capacity of increasing the bioavailability of phosphorus and potassium in the rhizosphere, and the ability of controlling *Fusarium* spp (Zapata et al., 2024). Similarly, in our earlier research, we isolated *A. tubingensis* HZ123, which showed potential as a PGPF by improving eggplant growth and soil fertility (Li et al., 2021).

**Table 5:** Mechanism of plant growth promotion by PGPF on different plants.

PGPF	Plants	Mechanisms	References
*Aspergillus*			
*A. brunneoviolaceus* (HZ23)	Pakchoi	Improved soil properties (especially phosphorus), rhizosphere bacterial community structure, and metabolites.	Li et al., 2023
*A. caespitosus* (DS-3)	Fenugreek	Produced IAA. Induced desirable physiological properties (with higher protein content, carbohydrate content, total phenolic content and antioxidant activity than control).	Thakor et al., 2023
*A. elegans*	Cucumber	Had a zinc solubilizing capacity.	Sidhoum et al., 2024
*A. falvus*	Wheat	Induced plant resistance against wilt disease.	El-Maraghy et al., 2020
*A. niger* (9-P)	Common bean	Produced IAA, siderophores, and hogh phosphorus solubilizing activity. Synthesized ACC deminase.	Galeano et al., 2021
*A. tubingensis*	Common bean	Increased the bioavailability of phosphorus and potassium in the rhizosphere. Promoted nutrition, chlorophyll content, and biometric parameters of plant. Controlled *Fusarium* spp.	Zapata et al., 2024
*Penicillium*			
*P. buchwaldii*	Tomato	Enhanced tomato immunity against root-knot nematodes.	Attia et al., 2025
*P. chrysogenum* (PenC-JSB9)	Pearl millet	Enhanced seed germination, root length, and shoot length. Induced resistance to downy mildew.	Murali et al., 2013
*P. citrinum*	Wheat	Induced plant resistance against a pathogen.	El-Maraghy et al., 2020
*P. janthinellim*	Melon	Elicited IAA. Induced resistance against stem rot caused *R. solani*.	Murali et al., 2021
*P. limosum* (AK-7)	/	Exhibited antifungal activities.	Basavarajappa et al., 2023
*P. olsonii* (A3)	Tobacco	Stimulated tobacco plant growth, enhanced its salt tolerance, and reduced by half the required chemical fertilizer inputs in a hydroponic farming system.	Tarroum et al., 2022
*P. oxalicum* (UOMPGPF16)	Pearl millet	Enhanced seed germination and seedling vigor. Enhanced NPK uptake. Induced resistance against downy mildew disease.	Murali and Amruthesh, 2015
*P. simplicissimum* (GP17-2)	Cucumber	Involved multiple defense mechanisms.	Hossain et al., 2007
*Talaromyces*			
*T. purpureogenus* (M13026-2)	Cucumber	Colonized in the rhizosphere soil stably. Phosphorus dissolving and siderophores production.	Zhao et al., 2021
*T. islandicus*	Common bean	Increased the bioavailability of phosphorus and potassium in the rhizosphere. Promoted nutrition, chlorophyll content, and biometric parameters of common bean. Controlled *Fusarium* spp.	Zapata et al., 2024
*T. veerkampii*	**/**	**/**	**/**
*T. wortmannii* (FS2)	Cabbage	Promoted growth and induced resistance.	Yamagiwa et al., 2011

### 4.2 Effect of PGPF on rice growth

The novelty in this study is the significant enhancement of rice growth and yield by *T. veerkampii*. Notably, there is limited literature on the agricultural application of *T. veerkampii*. In other words, our research further confirms the plant growth-promoting capabilities of *A. tubingensis* and *P. limosum*, isolated from non-grain soils, and highlights the potential of *T. veerkampii* to improve non-grain soils and promote crop growth. These findings provide valuable fungal isolates for the development of novel fungi-based products, such as bio-organic fertilizers and bio-amendments, grounded in PGPF. Furthermore, this study lays a theoretical foundation for improving non-grain soil conditions and enhancing crop growth. Further research should be conducted to elucidate the growth-promoting mechanisms of these fungal isolates.

### 4.3 Change of soil properties contributes to rice growth by PGPF

Among all 15 PGPF isolates, isolates TL-B31f (identified as *A. tubingensis*) and FY-R41f (identified as *T. veerkampii*) with the best growth-promoting properties were selected for further study. In fact, results showed that the soil nutrient elements could be significantly improved by inoculation of isolates TL-B31f and FY-R41f, in particular, the AP of 27.33 and 28.47 mg/kg, respectively, were greater than that of the control (19.17 mg/kg). Aligning with this result, our previous studies indicated that the application of *A. brunneoviolaceus* isolate HZ23 caused a significant increase in AP compared to the control (854.09 mg/kg *vs* 38.82 mg/kg) (Li et al., 2023). Phosphate application in farmland could enhance crop yield, while to increase phosphate use efficiency, and improve the fertility of low phosphate soils, which makes it a promising approach for sustainable agriculture (Li et al., 2015; Wang et al., 2024). Therefore, it can be inferred that isolates TL-B31f and FY-R41f may have a great effect on the improvement of the soil quality of non-grain cultivated land.

### 4.4 Change of microbial communities contributes to rice growth by PGPF

According to previous research, the effect of PGPF on various aspects of plant growth was variable, depending mostly on their successful survival, colonization, growth, and efficient adaptation to environmental conditions. Root colonization was an important strategy of PGPF for plant growth promotion. Upon colonization of the host plant, PGPF can change the microbial community features in the soil, thereby improving the plants’ well-being and productivity through a beneficial association with plants (Adedayo and Babalola, 2023). After 55 days of inoculation, isolates TL-B31f and FY-R41f had an obvious effect in increasing the richness of fungal communities, while the bacterial communities were not significantly changed. In all treatments, Pseudomonadota, Chloroflexota, Acidobacteriota, Bacteroidota, *OLB13*, *Luteitalea*, *Aggregatilinea*, *Subgroup 10*, *Vibrionimonas*, *MND1*, *Gemmatimonas*, and *Sphingomonas* were the main bacteria, while Ascomycota, Basidiomycota, Mucoromycota, *Botryotrichum*, *Mortierella*, *Fusarium*, *Triangularia*, and *Phlebiopsis* were the main fungi. In fact, more attention should be paid to Pseudomonadota, Chloroflexota, Acidobacteriota, *Sphingomonas*, Ascomycota, Basidiomycota, *Botryotrichum*, *Mortierella*, *Fusarium*, *Triangularia*, and *Phlebiopsis*. Pseudomonadota can mediate nutritional and growth-promotional activities for sustainable food security (Sah et al., 2021). Chloroflexota members may play a role in the nitrogen cycle, thereby improving the nitrogen removal performance in anammox bioreactors and activated sludge systems (Bovio-Winkler et al., 2024). Acidobacteriota is often an important contributor to the nutrient cycling system in soil microhabitats (Zhu et al., 2022a). *Sphingomonas* possess multifaceted functions ranging from the remediation of environmental contaminants to the production of highly beneficial phytohormones (Asaf et al., 2020). Ascomycota and Basidiomycota have the ability to degrade soil organic matter by producing cellulolytic enzymes (Manici et al., 2024). Mucoromycota can produce lipids, ethanol, organic acids, pigments, and enzymes. Thus, it has been considered a powerful cell factory for modern biorefinery (Dzurendova et al., 2022). *Botryotrichum* can produce some secondary metabolites that are unfavorable for plant growth (Elkhateeb and Daba, 2022). *Mortierella* has the ability to increase nutrient uptake efficiency, which has a positive effect on crop protection against adverse conditions, and reduces the use of chemical fertilizers and pesticides (Ozimek and Hanaka, 2020). *Fusarium* can cause severe economic damage in different agricultural productions such as rice, wheat, potato, etc (Ekwomadu and Mwanza, 2023). *Triangularia* often grows as saprophytes in the ground among leaf litter or in association with plant roots (Huang et al., 2021). *Phlebiopsis* has been used as a biocontrol agent against *Heterobasidion annosum* (Terhonen et al., 2013).

Compared with the control, the RAs of all these microbes were changed by the application of isolates TL-B31f and FY-R41f to potentially increase soil health and decrease rice morbidity. It was particularly worth mentioning that the RAs of *Aspergillus* were significantly increased by 264.68% in At-TLB31f treatments, while *Talaromyces* were significantly increased by 1328.35% in Tv-FYR41f treatments. In addition, RDA and correlation network analysis of microbes with soil properties indicated that SOM was significantly positively correlated with *Ellin6067*, *Lysobacter*, and *Phaeodactylibacter*; AP was significantly positively correlated with *Acidiferrimicrobium* and *Mortierella*, but was significantly negatively correlated with *Curvularia*; TP was significantly positively correlated with *Phlebiopsis* and *Hyphodermella*; pH was significantly negatively correlated with *Mortierella* and *Aspergillus*, but was significantly positively correlated with *Talaromyces.* In agreement with the results of this study, Guo et al. (2021) showed that SOC, TN, and TP were the most important factors explaining variations in the bacterial community structure. Li et al. (2023) reported that the composition of bacterial communities in packchoi rhizosphere soil was affected significantly by AP, pH, OMC, and TN. Arunrat et al. (2023) found that soil bacterial community was strongly influenced by organic matter and organic carbon in Maize field. The growth of microbes in bayberry rhizosphere soil was affected by many environmental factors, including pH, OMC, AP, and exchangeable magnesium (Ren et al., 2021). Taken overall, compared to the control, the microbial community composition of the two PGPF isolates treatments was reconstructed with an increase or decrease of some specific microbes, and soil properties might play important roles in influencing the growth of microbial communities to modulate rice growth.

## 5 Conclusions

Overall, 15 PGPF isolated from soils of different non-grain converted land exhibited great ability to solubilize phosphate, to secrete siderophore and to produce IAA, while the effect varied with different fungal strains. Furthermore, all 15 fungal isolates were separated into six distant clades including *A. brunneoviolaceus*, *A. tubingensis*, *P. oxalicum*, *P. limosum*, *T. purpureogenus* and *T. veerkampii* through morphological identification and phylogenetic analysis. Notably, our results indicated that both *A. tubingensis* and *T. veerkampii* had significant growth-promoting effects on rice. Based on microbial communities’ analysis of rice root-zone soil after 55 days of inoculation, our findings further highlight their potential to be developed into novel microbial formulations, such as biofertilizers, aimed at improving soil conditions and enhancing crop growth in non-grain production lands.

## Data Availability

The datasets presented in this study can be found in online repositories. The names of the repository/repositories and accession number(s) can be found in the article/supplementary material.
